# Bacterial Adhesion in Pediatric Crowns: A Systematic and Meta-Analytical Review

**DOI:** 10.7759/cureus.65282

**Published:** 2024-07-24

**Authors:** Neshkumar K L S, Kavitha Ramar

**Affiliations:** 1 Pediatric and Preventive Dentistry, SRM (Sri Ramaswamy Memorial) Kattankulathur Dental College and Hospital, Chennai, IND; 2 Pedodontics and Preventive Dentistry, SRM (Sri Ramaswamy Memorial) Kattankulathur Dental College and Hospital, Chennai, IND

**Keywords:** oral microbiology, microbial adhesion, pediatric preventive dentistry, stainless steel crowns, systematic review and meta analysis

## Abstract

Stainless steel crowns (SSC) have long been the standard choice due to their durability, ease of use, and cost-effectiveness. Other esthetic crowns, known for their superior esthetic properties and biocompatibility, have emerged as a popular option in recent years. A comprehensive literature search was conducted across multiple databases, including PubMed, Scopus, and Cochrane Library, up to June 2024. The population under study was primary teeth, with the intervention being the application of aesthetic crowns. These were compared against SSC, with the primary outcome being the level of microbial adhesion on the crowns in primary teeth. Data extraction and quality assessment were performed independently by two reviewers. Meta-analyses were conducted using random-effects models to estimate pooled differences in microbial adhesion levels. A total of five studies were included in the review. The meta-analysis revealed a statistically significant difference in microbial adhesion between esthetic and SSC, with esthetic crowns showing lower levels of bacterial colonization. Specifically, the mean difference in colony-forming units (CFU). Esthetic crowns exhibit significantly lower microbial adhesion compared to SSC in pediatric patients, suggesting a potential advantage in reducing the risk of secondary caries and other microbial-related complications. However, further long-term clinical studies are warranted to confirm these results and assess their clinical implications.

## Introduction and background

Primary teeth are essential for children's growth and development. The need to maintain primary teeth until their permanent successors appear has driven the creation of diverse restorative materials and techniques. Dental caries in young children is often caused by poor eating habits, continuous nighttime breastfeeding, and poor oral hygiene [1]. When a caries problem occurs, dentists need to remove the lesion and fill it up, and the most commonly used material in recent years is resin. However, the resin restoration technique is often prone to secondary dental caries, resin or tooth fracture, postoperative sensitivity, and discoloration after long-term follow-up. That’s why dental crowns are often needed for dental caries in children [2]. Recent innovations in esthetic crowns, such as those made from zirconia, have significantly improved the appearance and durability of restorations, providing a more natural and long-lasting solution for maintaining children's dental health.

Stainless-steel crowns (SSC) are considered the first choice for the repair of severely damaged primary teeth and have been one of the most effective and efficient methods of tooth restoration in pediatric dentistry since Humphrey et al. first used them on pediatric patients in 1950 [3]. SSC has become an invaluable restorative technique for the treatment of badly broken-down primary teeth. The superiority and durability of SSC over multi-surface amalgam and other restorations in primary dentition have been documented in the literature [4]. Despite many advantages, the metal appearance of these crowns is unpleasant to parents and children. They prefer tooth-colored restorations to silver-colored fillings, regardless of the location of the restorations. Inflammation of the surrounding gingival tissue is also a problem frequently associated with SSC. The incidence of gingivitis has been reported to be higher around poorly fitting crowns than around crowns considered to be well adapted.

In the selection of dental crowns, SSC has been proven to be a material with high stability [5,6]. In contrast, resin crowns or pre-veneered SSC are more prone to embrittlement [7,8]. In the long-term follow-up results, SSC treatment still had a high success rate, regardless of the sealing material used. Due to the unnatural color of SSC, there are still other options for the anterior tooth crowns or to modify the SSC. Therefore, the present systematic review study aims to explore the literature on the comparison of SSC and other aesthetic crowns and analyze the results regarding their microbial adhesions.

## Review

Methods

The Preferred Reporting Items for Systematic Reviews and Meta-Analyses (PRISMA) guidelines [9] were followed throughout the processing stages of this study, which were performed according to the Cochrane Handbook for Systematic Reviews of Interventions [10]. The protocol of this review was registered as CRD42024539626 in the International Prospective Register of Systematic Reviews (PROSPERO) (https://www.crd.york.ac.uk/prospero).

The population for this study consists of primary teeth. The intervention involves using SSC, which is compared to other types used in pediatric dentistry. The primary outcome of this study is the assessment of microbial adhesion on two kinds of crowns in primary teeth.

Eligibility Criteria

For this systematic review, we established stringent inclusion and exclusion criteria to ensure the selection of relevant and high-quality studies. Our inclusion criteria included randomized controlled trials (RCTs), cross-sectional studies, and clinical trials that examined the impact of microbial adhesion on primary teeth, specifically focusing on SSC and other aesthetic crowns. The review targeted research directly addressing microbial adhesion, a crucial factor in the success and longevity of dental crowns in pediatric dentistry.

We excluded in vitro studies, as they lack the clinical relevance needed to understand microbial adhesion in a natural oral environment. Studies involving different types of restorations, such as fillings, were also excluded to maintain a clear focus on crowns. Additionally, we did not consider research on microleakage in crowns, as it relates more to the seal and longevity of the crowns than microbial adhesion. We also excluded studies on other types of crowns and those involving crowns on permanent teeth to ensure the review remained specific to primary teeth, which have unique anatomical and physiological characteristics influencing microbial adhesion.

Outcomes

The primary outcome of this systematic review is to evaluate and compare the level of microbial adhesion on zirconia crowns versus SSC used in primary teeth. This involves assessing the quantity and type of microbial colonies that adhere to the surfaces of these crowns over time, which directly impacts the risk of dental caries and other oral health issues. Specific outcomes measured include the number of colony-forming units (CFU) of bacteria adhering to each type of crown and the identification of specific bacterial species, such as *Streptococcus mutans*, known to be associated with dental caries.

Endpoint

The endpoint would be the comparative quality of microbial adhesion levels on SSC and esthetic crowns in pediatric patients following their placement.

Search Strategy and Study Selection

Two reviewers (NK and KR) independently conducted a literature search until June 2024. For related papers, a comprehensive literature search was performed using eight search strategies: PubMed, Web of Science, Scopus, Google Scholar, Lilacs, Cochrane, EBSCO (Elton B. Stephens Company), and Clinical Key. The keywords and MeSH (Medical Subject Headings) terms used were (((((((((((Microbial adhesion) AND (Zirconia crown)) OR (Esthetic crown)) AND (Stainless steel crown)) OR (SSC)) OR (Pre veneered crown)) OR (Metal crown)) AND (Primary teeth). This search was accompanied by a manual search for additional literature in relevant references.

The search results were reviewed, and studies were chosen according to specific selection criteria. Publications were initially excluded based on their titles, followed by their abstracts, and lastly, the full texts that were obtained. The bibliographic references of these publications were manually examined to uncover any other relevant studies that might have been missed in the initial phases. The data were then extracted and compiled into an electronic spreadsheet for further analysis.

The full-text articles were then independently reviewed by the same two reviewers. During this stage, studies were meticulously examined to confirm that they met all inclusion criteria and to extract detailed information about their methodologies, interventions, comparisons, and outcomes. Any discrepancies between the reviewers were resolved through discussion and consensus, with a third reviewer consulted if necessary.

Quality Assessment

In this systematic review, we thoroughly assessed the risk of bias for each included study to ensure the reliability and validity of our findings. RCTs were evaluated using the Cochrane Risk of Bias Tool, which examines various domains such as selection bias, performance bias, detection bias, attrition bias, and reporting bias. Each domain was scrutinized to identify any potential biases that could affect the study outcomes.

The included studies were individually randomized parallel-group trials, with ‘SSC’ considered as the experimental group and the ‘Other types of crowns’ as the comparator. The risk of bias assessment was evaluated by two reviewers (NK and KR) using the Cochrane Collaboration Guidelines, a revised tool to assess the risk of bias in randomized trials (ROB 2.0). This instrument consists of five main domains and an overall risk of bias domain [11]. The five main domains are the randomization process, deviations from the intended intervention, missing outcome data, measurement of the outcome, and selection of reported results. Each of the included articles (five in total) was categorized as low risk if all the signaling questions were met, high risk if one or more criteria were not met, and unclear risk of bias (or some concerns) if one or more criteria were partly met or had insufficient information. The overall risk of bias was determined by the highest risk identified in any of the assessed domains. Any disagreements were resolved by the first reviewer (NK) [12]. The domain-level judgments for each study (traffic light plot) and the distribution of risk-of-bias judgments within each bias domain (summary bar plot) were generated to represent the risk of bias assessment graphically using Robvis.

Statistical Analysis

Data on the outcomes were extracted from each study and initially entered into Microsoft Excel (Microsoft Corporation, Redmond, Washington, United States). The statistical analysis was conducted using Review Manager version 5.4.1 (RevMan; The Cochrane Collaboration, 2020). The chi-square test and the I^2^ test were used to calculate the heterogeneity among the studies. For an expected outcome of continuous data type, an inverse variance statistical method along with a random effects analysis model was employed, and the effects estimate measure was expressed as mean difference (MD) with totals, subtotals, and a 95% confidence interval. The pooled effect measure (overall effect) was estimated using the Z test. The level of significance is determined at p<0.05. For graphical presentations of the results, forest plots and funnel plots were created.

Results

Literature Search

From the initial search, a total of 3987 articles were identified. After removing duplicates and excluding studies, 3828 unique records remained for screening.

In the first phase of screening, the titles and abstracts of these 3828 records were reviewed independently by two reviewers (NK and KR) to assess their relevance based on the predefined inclusion and exclusion criteria. This screening process resulted in the exclusion of 1190 studies, leaving 153 full-text articles for further assessment.

**Figure 1:**
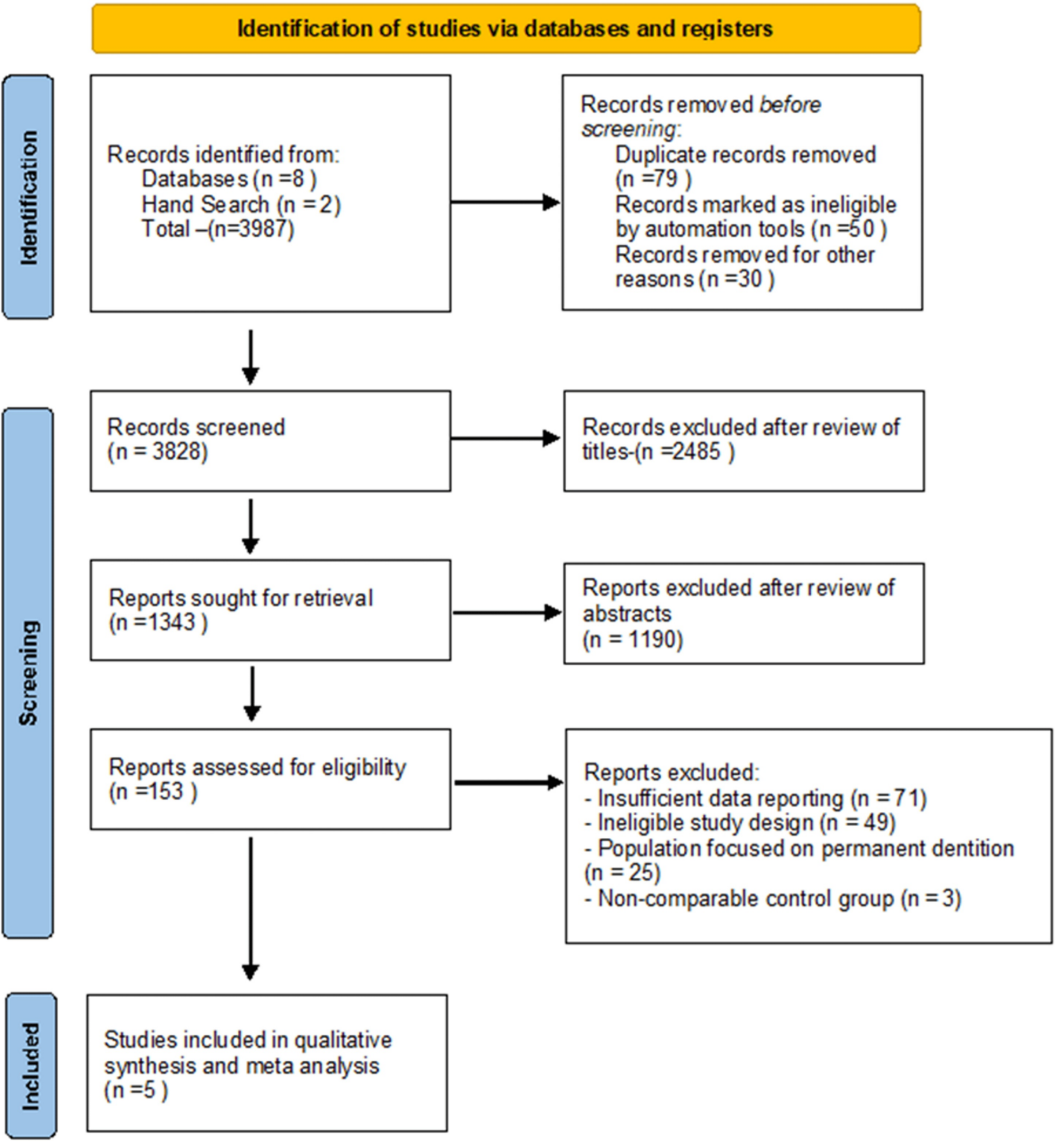
PRISMA flow diagram of the literature research process and results PRISMA: Preferred Reporting Items for Systematic Reviews and Meta-Analyses

This thorough evaluation led to the exclusion of 148 studies, primarily due to reasons such as inadequate data reporting, inappropriate study design, or failure to meet the inclusion criteria such as another type of restoration, in-vitro studies, and microleakage of crowns. Ultimately, five studies were deemed eligible and included in the qualitative synthesis (Figure 1, Table 1).

**Table 1:** Characteristics of the included studies SSC: stainless steel crown; RCT: randomized controlled trial

Title	Author	Outcome	Comparison	Significant	Type of Study	Year
Comparative study on the microbial adhesion of pre-veneered and SSC	AlShaibah et al. [13]	Count of *Streptococcus mutans*	Pre-veneered crown and SSC	Yes	In vivo	2012
Assessment of microbial adhesion to zirconia and stainless steel crowns in primary molars	Wakwak et al. [14]	Count of *Streptococcus mutans* and *Lactobacillus*	Zirconia and SSC	Yes	RCT	2019
Evaluation of adhesion of *Streptococcus mutans*, plaque accumulation on zirconia and stainless steel crowns, and surrounding gingival inflammation in primary molars: randomized controlled trial	Mathew et al. [15]	Count of *Streptococcus mutans*	Zirconia and SSC	Yes	RCT	2020
Comparative evaluation of adhesion of *S. mutans* on Figaro crowns in primary molars-a randomized clinical trial	Subramanian et al. [16]	Count of *Streptococcus mutans*	Figaro crowns and SSC	Yes	RCT	2022
Evaluation of *Streptococcus mutans* colonization and oral hygiene status in primary molars restored with two different crowns: a randomized clinical trial	Elizabeth et al. [17]	Count of *Streptococcus mutans*	Zirconia and SSC	Yes	RCT	2023

Quality Assessment

**Figure 2:**
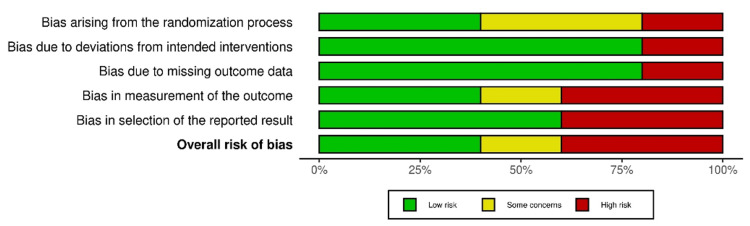
Summary of the risk of bias for each domain among the included studies.

**Figure 3:**
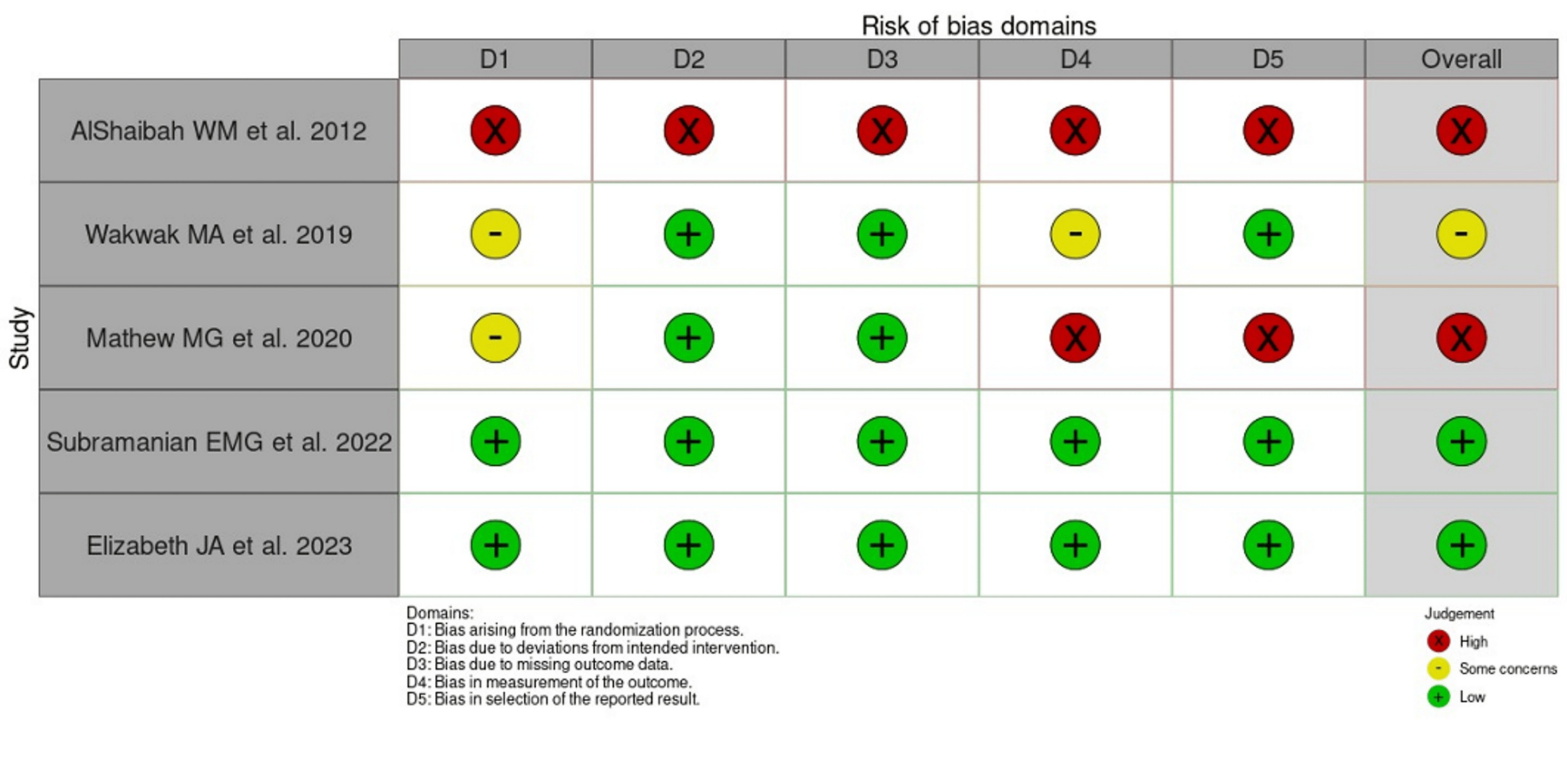
Risk of bias for each study via the revised RoB tool for randomized controlled trials. References: AlShaibah et al. [13], Wakwak et al. [14], Mathew et al. [15], Subramanian et al. [16], Elizabeth et al. [17]

A risk of bias assessment was conducted for the five included articles. The studies by Subramanian et al. [16] and Elizabeth et al. [17] demonstrated a very low level of risk of bias. In contrast, the study by Wakwak et al. [14] exhibited a minimal level of risk of bias. The remaining two studies, however, showed a high level of risk in several aspects, including randomization, intervention, outcome data reporting, and the measurement of outcomes (Figures 2, 3).

Outcomes

**Figure 4:**
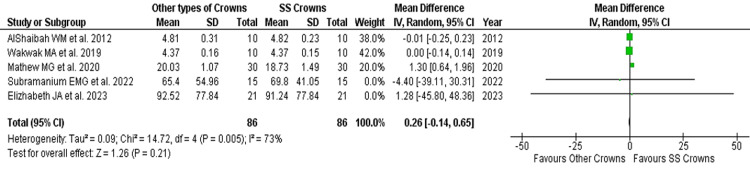
Mean difference and weights of Streptococcus mutans (CFU/ml) before crown placement with forest plot References: AlShaibah et al. [13], Wakwak et al. [14], Mathew et al. [15], Subramanian et al. [16], Elizabeth et al. [17]

**Figure 5:**
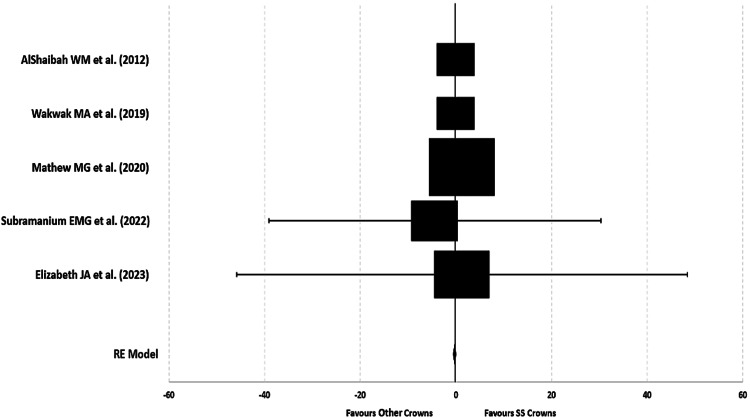
Forest plot of mean difference of Streptococcus mutans (CFU/ml) before crown placement AlShaibah et al. [13], Wakwak et al. [14], Mathew et al. [15], Subramanian et al. [16], Elizabeth et al. [17]

Microbial adhesion count in both crowns before placement: The pooled MD in a random model analysis of *S. mutans* colony-forming units per milliliter (CFU/ml) on teeth before crown placement was 0.26 (pooled 95%CI: -0.14, 0.65) with almost no effect size and non-significant (p > 0.05). Two studies showed the MD of *S. mutans* to be higher in the control group (SSC), and one study favored the experimental group (other types of crowns) with a broader 95%CI. Whereas, another two studies showed no possible MD (no effect) with the least 95%CI. The I2 statistic of *S. mutans* microbial adhesion before crown placement shows substantial heterogeneity of 73% (Tau2 = 0.009; ꭓ2 = 14.72, p = 0.005, significant). The test for overall effect non-significantly favors none of the groups (Z = 1.26; p = 0.21, not significant) (Table 2, Figures 4, 5).

**Table 2:** Mean difference and weights of Streptococcus mutans (CFU/ml) before crown placement Heterogeneity: Tau2 = 0.09; Chi2 = 14.72, df = 4 (p = 0.005); I2 = 73% Test for overall effect Z = 1.26 (p = 0.21)

Study or Subgroup	Other Types of Crowns			Stainless Steel Crowns			Weight (%)	Mean Difference
	Mean	SD	Total	Mean	SD	Total		IV, Random (95% CI)
AlShaibah et al. (2012) [13]	4.81	0.31	10	4.82	0.23	10	38	-0.01 (-0.25, 0.23)
Wakwak et al. (2019) [14]	4.37	0.16	10	4.37	0.15	10	42	0.00 (-0.14, 0.14)
Mathew et al. (2020) [15]	20.03	1.07	30	18.73	1.49	30	20	1.30 (0.64, 1.96)
Subramanium et al. (2022) [16]	65.4	54.96	15	69.8	41.05	15	0	-4.40 (-39.11, 30.31)
Elizabeth et al. (2023) [17]	92.52	77.84	21	91.24	77.84	21	0	1.28 (-45.80, 48.36)
Total (95% CI)			86			86	100	0.26 (-0.14, 0.65)

**Figure 6:**
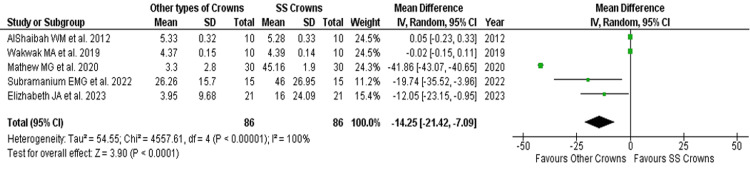
Mean difference and weights of Streptococcus mutans (CFU/ml) after crown placement References: AlShaibah et al. [13], Wakwak et al. [14], Mathew et al. [15], Subramanian et al. [16], Elizabeth et al. [17]

**Figure 7:**
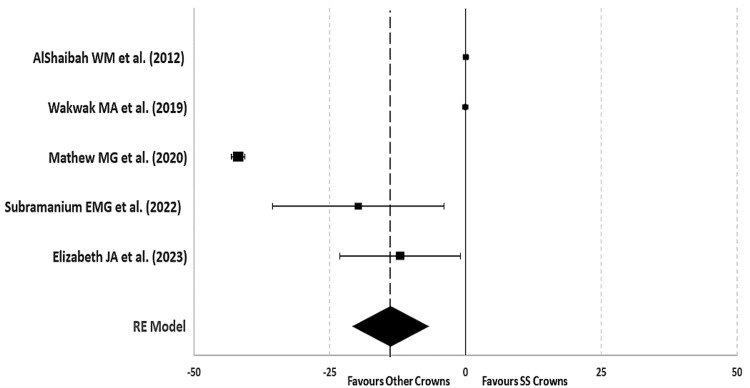
Forest plot of mean difference of Streptococcus mutans (CFU/ml) after crown placement References: AlShaibah et al. [13], Wakwak et al. [14], Mathew et al. [15], Subramanian et al. [16], Elizabeth et al. [17]

Microbial adhesion count in both crowns after placement: After crown placement, the combined microbial adhesion of *S. mutans* was significantly higher in the experimental group (other types of crowns), with a pooled MD CFU/ml in a random model analysis of -14.25 (pooled 95%CI: -21.42, -7.09; p<0.05). The test for overall effect showed higher *S. mutans* CFU/ml in the experimental group (Z = 3.90; p<0.0001, significant) than SSC. Three studies individually favored the experimental group with a broader 95%CI, and two studies showed no conceivable MD (no effect) with the least potential 95%CI. The statistically significant chi-square test for heterogeneity (Tau2 = 54.55; ꭓ2 = 4557.61, p<0.00001) suggests that there was considerable heterogeneity between and within the included studies (I^2^ statistic = 100%) (Table 3, Figures 6, 7).

**Table 3:** Mean difference and weights of Streptococcus mutans (CFU/ml) after crown placement Heterogeneity: Tau2 = 54.55; Chi2 = 4557.61, df = 4 (p<0.00001); I2 = 100% Test for overall effect Z = 3.90 (p<0.0001)

Study or Subgroup	Other Types of Crowns			Stainless Steel Crowns			Weight (%)	Mean Difference
	Mean	SD	Total	Mean	SD	Total		IV, Random (95% CI)
Waleed et al. (2012) [13]	5.33	0.32	10	5.28	0.33	10	24.5	0.05 (-0.23, 0.33)
Wakwak et al. (2019) [14]	4.37	0.15	10	4.39	0.14	10	24.5	-0.02 (-0.15, 0.11)
Mathew et al. (2020) [15]	3.3	2.8	30	45.16	1.9	30	24.3	-41.86 (-43.07, -40.65)
Subramanium et al. (2022) [16]	26.26	15.7	15	46	26.95	15	11.2	-19.74 (-35.52, -3.96)
Elizabeth et al. (2023) [17]	3.95	9.68	21	16	24.09	21	15.4	-12.05 (-23.15, -0.95)
Total (95% CI)			86			86	100	-14.25 (-21.42, -7.09)

Publication Bias

**Figure 8:**
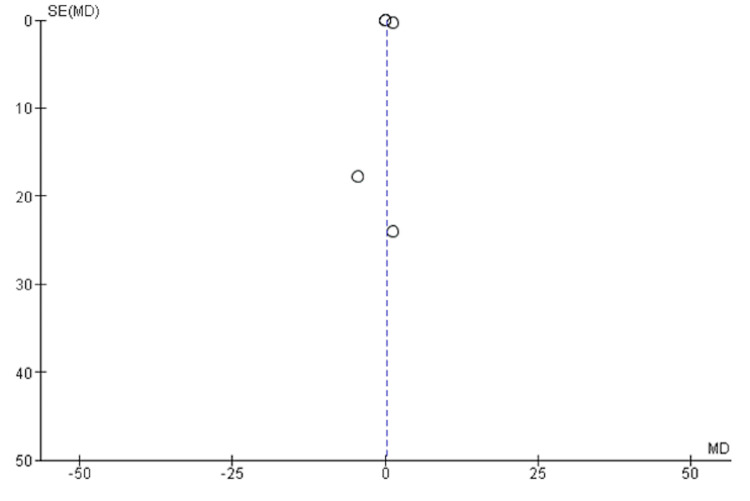
Funnel plot of mean difference of Streptococcus mutans (CFU/ml) before crown placement

**Figure 9:**
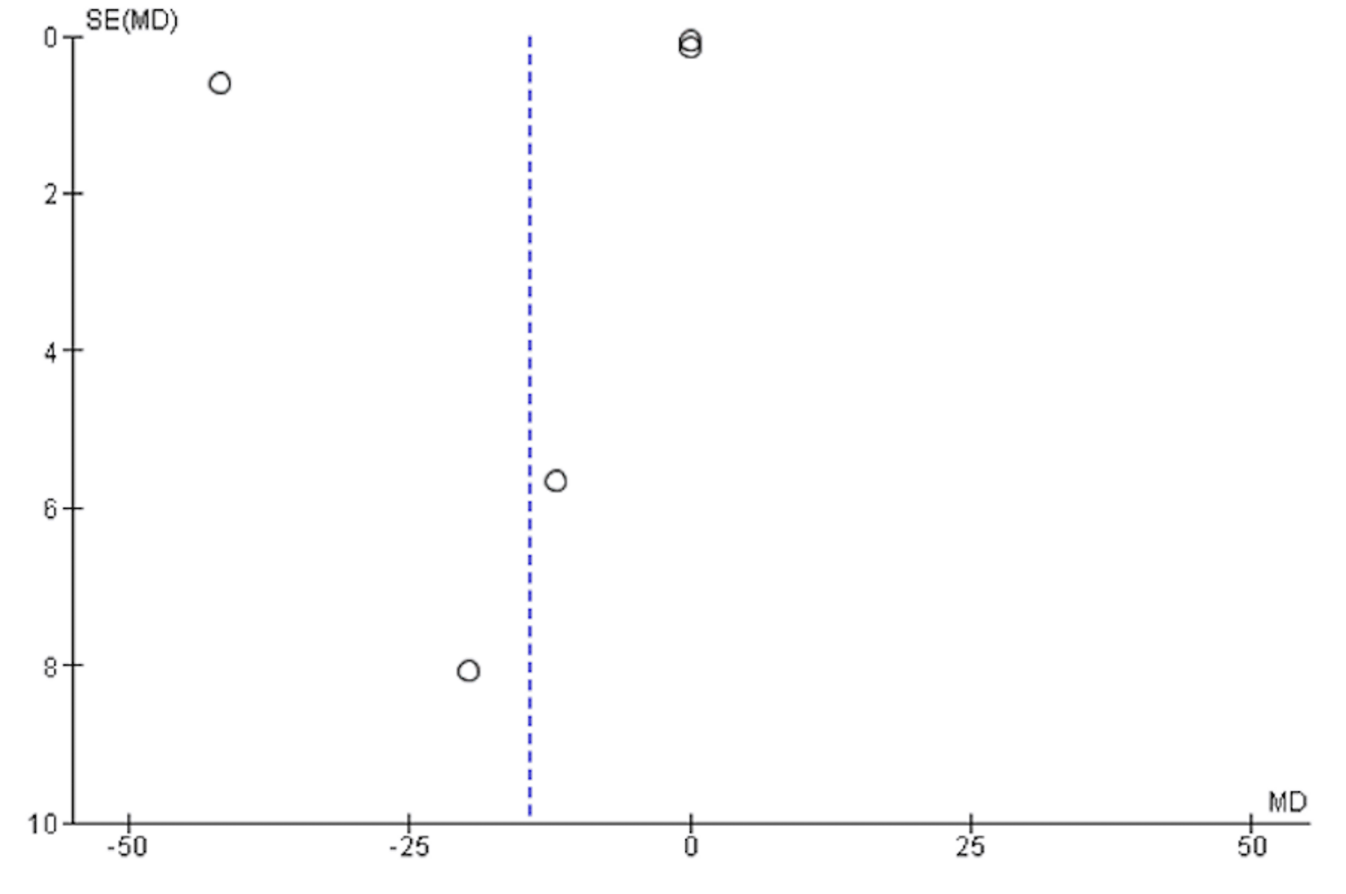
Funnel plot of mean difference of Streptococcus mutans (CFU/ml) after crown placement

A funnel plot was performed to assess for publication bias in all the studies included in the meta-analysis. The symmetrical distribution of effect estimates (MD) as observed against their standard errors in the funnel plot (Figure 8) indicates no publication bias. Whereas, the asymmetrical distribution of effect estimates in Figure 9 (*S. Mutans *microbial adhesion after crown placement) suggests that publication bias is likely to be a major issue in this meta-analysis.

Discussion

The oral cavity is a dynamic environment, constantly exposed to various substances like saliva, food particles, and oral biofilms along with their metabolites. These biofilms, comprising bacterial communities, adhere to teeth, dental materials, and both hard and soft tissues and are primarily responsible for dental caries and gingival inflammation in children [18]. *S. mutans* is a key microorganism associated with dental caries, as it facilitates the formation of cariogenic biofilms by producing a matrix rich in insoluble exopolysaccharides, particularly α 1,3-glucans. Consequently, this study focused on assessing the colonization of *S. mutans* on full-coverage restorations [19]. This systematic review aimed to evaluate the performance of crowns for primary teeth by analyzing data from eight studies that examined various clinical aspects, such as microbial adhesion and *S. mutans* colony formation around the crowns. The outcomes varied depending on the chemical composition of the material surfaces, which is crucial for bacterial colonization. Zirconia, known for its chemical stability and biocompatibility, demonstrated a minimal rate of component release [20]. 

This systematic review and meta-analysis provide a comprehensive evaluation of microbial adhesion on pediatric dental crowns, comparing SSC with other types of crowns. The findings indicate significant variability in microbial adhesion levels across different crown materials, underscoring the importance of material selection in pediatric dentistry.

Al-Shaibah et al. [13] offer essential insights into the comparative effectiveness of pre-veneered and SSC in pediatric dentistry. The lower microbial adhesion on pre-veneered crowns, demonstrated by reduced CFU counts and less biofilm formation observed through scanning electron microscopy (SEM), suggests that pre-veneered crowns are more effective in preventing microbial colonization compared to SSC. The clinical implications are substantial: opting for preferred crowns over stainless steel could potentially lower the incidence of caries and other microbial-related complications in pediatric patients, leading to better long-term outcomes, fewer follow-up treatments, and enhanced overall effectiveness and cost-efficiency of pediatric dental care. However, the study's focus on primary molars may limit the generalizability of the results to other types of teeth or age groups.

Wakwak et al. offer valuable insights into the comparative effectiveness of zirconia and SSC in preventing microbial adhesion in primary molars [14]. Their study shows that zirconia crowns have lower microbial adhesion, as evidenced by reduced CFU counts and less biofilm formation observed through SEM. This indicates zirconia is a more favorable material for pediatric dental restorations, reducing the risk of dental caries and periodontal disease. These findings have significant clinical implications: selecting zirconia crowns over stainless steel could potentially lower the incidence of secondary caries and other microbial-related complications in pediatric patients, resulting in better long-term outcomes and fewer follow-up treatments. 

Matthew et al. provide important insights into the performance of zirconia and SSC in pediatric dental care [15]. The study indicates that zirconia crowns are associated with significantly lower levels of *S. mutans* adhesion and plaque accumulation compared to SSC. Additionally, the reduced gingival inflammation observed around zirconia crowns underscores their potential for better periodontal health. These results are consistent with other studies showing zirconia's benefits in resisting microbial colonization and plaque formation. Zirconia's smooth surface and biocompatibility contribute to its lower tendency for microbial adhesion and plaque retention, which in turn reduces the risk of gingival inflammation. This is particularly important for pediatric patients, where maintaining oral hygiene can be challenging.

Subramanian et al. add valuable data to the expanding research on crown materials' effectiveness in pediatric dentistry [16]. Their findings reveal that *S. mutans* adhesion is significantly lower on Figaro crowns compared to SSC, highlighting the potential benefits of Figaro crowns in preventing dental caries in primary molars. These results align with broader dental materials research trends, where newer materials like Figaro crowns are engineered to possess enhanced anti-biofilm properties. The reduced microbial adhesion is attributed to the specific material composition and surface characteristics of Figaro crowns, including lower surface roughness and hydrophobic properties that deter microbial colonization. These findings support the use of Figaro crowns as a superior choice in pediatric dental restorations, promoting better preventive care and improved dental health outcomes for children. Integrating this evidence into clinical practice can help optimize treatment strategies and enhance the quality of pediatric dental care.

Elizabeth et al. provide valuable insights into the comparative effectiveness of two different crown materials in pediatric dentistry [17]. The finding that *S. mutans* colonization is significantly lower with zirconia suggests this new material has superior anti-microbial properties compared to traditional SSC. This reduction in microbial colonization is crucial for minimizing the risk of dental caries in children. These results are consistent with broader trends in dental material research, where newer materials are designed to enhance biocompatibility and reduce microbial adhesion. The clinical implications are significant: choosing zirconia over a traditional SSC could lead to better long-term oral health outcomes for pediatric patients, reducing caries and periodontal diseases, and decreasing the need for further dental interventions. However, the study has certain limitations, such as a follow-up period that may not fully capture the long-term performance of the crown materials and uncontrolled patient-specific factors like dietary habits, oral hygiene routines, and saliva composition variations, which could influence the results. The study also highlights the advantages of zirconia crowns in reducing *S. mutans* colonization and improving oral hygiene status in primary molars. 

To systematically evaluate microbial adhesion in esthetic crowns versus SSC for primary teeth, this study encountered a few limitations. The primary concern arises from the heterogeneity observed in the forest plot analysis, which suggests variability in study outcomes. While the conclusions from reviewed studies generally favored lower microbial adhesion in zirconia crowns compared to SSC, the forest plot indicated a high risk of bias with data skewed towards the left side, indicating potential overestimation or inconsistency in reported effects. This heterogeneity could stem from various sources, including differences in study designs, methodologies, and population characteristics across the included studies. Additionally, the risk of bias within individual studies, as suggested by the forest plot, raises concerns about the reliability and generalizability of the findings. Future systematic reviews should consider these limitations and strive for more uniform study designs and rigorous methodologies to enhance the robustness of conclusions regarding microbial adhesion in pediatric dental crown materials.

## Conclusions

This systematic review and meta-analysis indicate that zirconia crowns exhibit lower microbial adhesion compared to SSC in primary teeth. However, significant heterogeneity and a high risk of bias among the included studies limit the reliability and generalizability of these findings. Future research with standardized designs and rigorous methodologies is needed to confirm these results and provide clearer clinical guidance.
